# Degradation of Marine Algae-Derived Carbohydrates by Bacteroidetes Isolated from Human Gut Microbiota

**DOI:** 10.3390/md15040092

**Published:** 2017-03-24

**Authors:** Miaomiao Li, Qingsen Shang, Guangsheng Li, Xin Wang, Guangli Yu

**Affiliations:** 1 Shandong Provincial Key Laboratory of Glycoscience and Glycoengineering, School of Medicine and Pharmacy, Ocean University of China, Qingdao 266003, China; liaa99@163.com (M.L.); shangqingsen@163.com (Q.S.); 2 Laboratory for Marine Drugs and Bioproducts of Qingdao National Laboratory for Marine Science and Technology, Qingdao 266237, China; 3 DiSha Pharmaceutical Group, Weihai 264205, China; gsli-87@163.com; 4 State Key Laboratory of Breeding Base for Zhejiang Sustainable Pest and Disease Control and Zhejiang Key Laboratory of Food Microbiology, Academy of Agricultural Sciences, Hangzhou 310021, China

**Keywords:** carrageenan, agarose, alginate, oligosaccharides, *Bacteroides xylanisolvens*, *Bacteroides ovatus*, *Bacteroides uniforms*

## Abstract

Carrageenan, agarose, and alginate are algae-derived undigested polysaccharides that have been used as food additives for hundreds of years. Fermentation of dietary carbohydrates of our food in the lower gut of humans is a critical process for the function and integrity of both the bacterial community and host cells. However, little is known about the fermentation of these three kinds of seaweed carbohydrates by human gut microbiota. Here, the degradation characteristics of carrageenan, agarose, alginate, and their oligosaccharides, by *Bacteroides xylanisolvens*, *Bacteroides ovatus*, and *Bacteroides uniforms*, isolated from human gut microbiota, are studied.

## 1. Introduction

Marine carbohydrates contain a great deal of polysaccharides and oligosaccharides, some of them have been used as food additives for a long time, such as carrageenan, agarose, and alginate; all play an important role in Asian food, however, these marine carbohydrates cannot be digested by the enzymes encoded by the human genome. By now, most of the reported enzymes responsible for digesting marine carbohydrates are from ocean bacteria [1,2,3]; however, recent studies showed that the genes encoding porphyranase and agarase, which, respectively, cleave the algal polysaccharides porphyran and agarose, were found in *Bacteroides plebeius*, isolated from the microbiota of Japanese individuals [4,5]. This means that the human gut microbiota assist humans in utilizing marine carbohydrates that could not be digested by humans. 

Genome analysis showed that human gut microbiota contained abundant and a variety of carbohydrate-active enzymes, which endow us with metabolic abilities that compensate for the paucity of glycoside hydrolases and polysaccharide lyases encoded by our genome [6]. However, the human gut microbiota is a massive colony, with 10^14^ microbes, including 1000 to 1150 phylotypes [7]; it is hard to assign the degradation of carbohydrates to specific bacterium. Notably, for marine carbohydrates, despite the fact that they have been used as food additives for a long time, little is known regarding their degradation and utilization by specific bacterium from human gut microbiota. 

Our previous results showed the degradation of agaroligosaccharides [8], alginate, and alginate oligosaccharides [9] by Chinese gut microbiota*.* Here, we report the degradation of several kinds of marine carbohydrates (carrageenan oligosaccharides, agarose, alginate, and alginate oligosaccharides) by several Bacteroidetes isolated from Chinese individuals’ feces, and analyze the structure of degradation products and the enzymes responsible for degrading marine carbohydrates. The results showed that *Bacteroides uniforms* L8 could degrade agarose (AP) completely, and the major enzyme secreted was β-agarase. The enzyme produced by isolate 38F6 (*Bacteroides xylanisolvens* and *Escherichia coli*), which degrades κ-carrageenan oligosaccharides, is β-carrageenase; for *Bacteroides ovatus* G19, based on its digestion pattern of alginate (Alg), guluronic acid oligosaccharides (GO), and mannuronic acid oligosaccharides (MO); the enzymes contain both α-1,4-guluronanlyase and β-1,4-mannuronanlyase.

## 2. Results and Discussion

### 2.1. Chemical Structures of the Products Generated by the Degradation of κ-Carrageenan Oligosaccharides by B. xylanisolvens and E. coli 

Isolate 38F6 that degraded κ-carrageenan oligosaccharides was identified as two kinds of bacterium: *B. xylanisolvens* and *E. coli*. We attempted to purify *B. xylanisolvens* from the complex isolate, but the degrading ability of the single *B. xylanisolvens* is much weaker than that of the complex [10]. Then, 38F6 was used to inoculate neocarratetraose (NK-DP4), neocarrahexaose (NK-DP6), and carraheptadecaose (K-DP17). κ-carrageenan is a linear polysaccharide composed of repeating disaccharides of (1→3)-4-SO_4_-β-d-galactose (G4S) and (1→4)-3,6-anhydro-α-d-galactose (A). The sequence of NK-DP4, NK-DP6 and K-DP17 are A-G4S-A-G4S, A-G4S-A-G4S-A-G4S, and (-G4S-(A-G4S)_n_-, *n* = 8).

**Figure 1:**
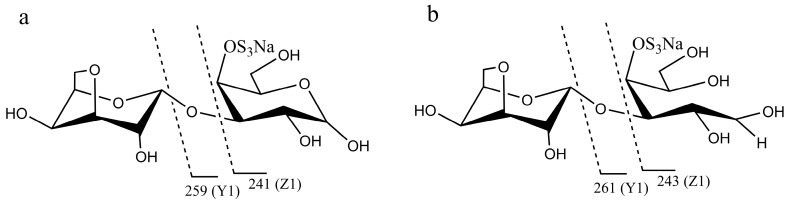
Diagram of fragments generated from electrospray ionization collision-induced-dissociation mass spectrometry (ESI-CID-MS^2^) of neocarrabiose and neocarradiitol. (**a**) neocarrabiose; (**b**) neocarradiitol.

**Figure 2:**
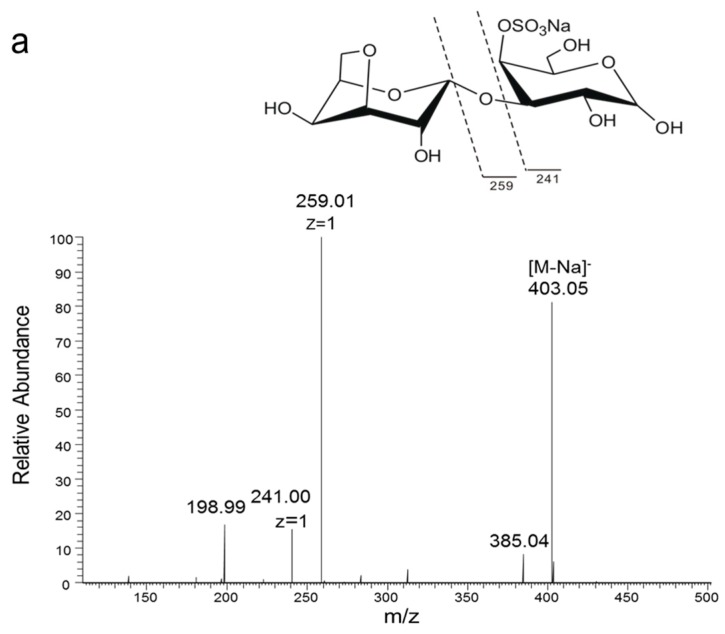

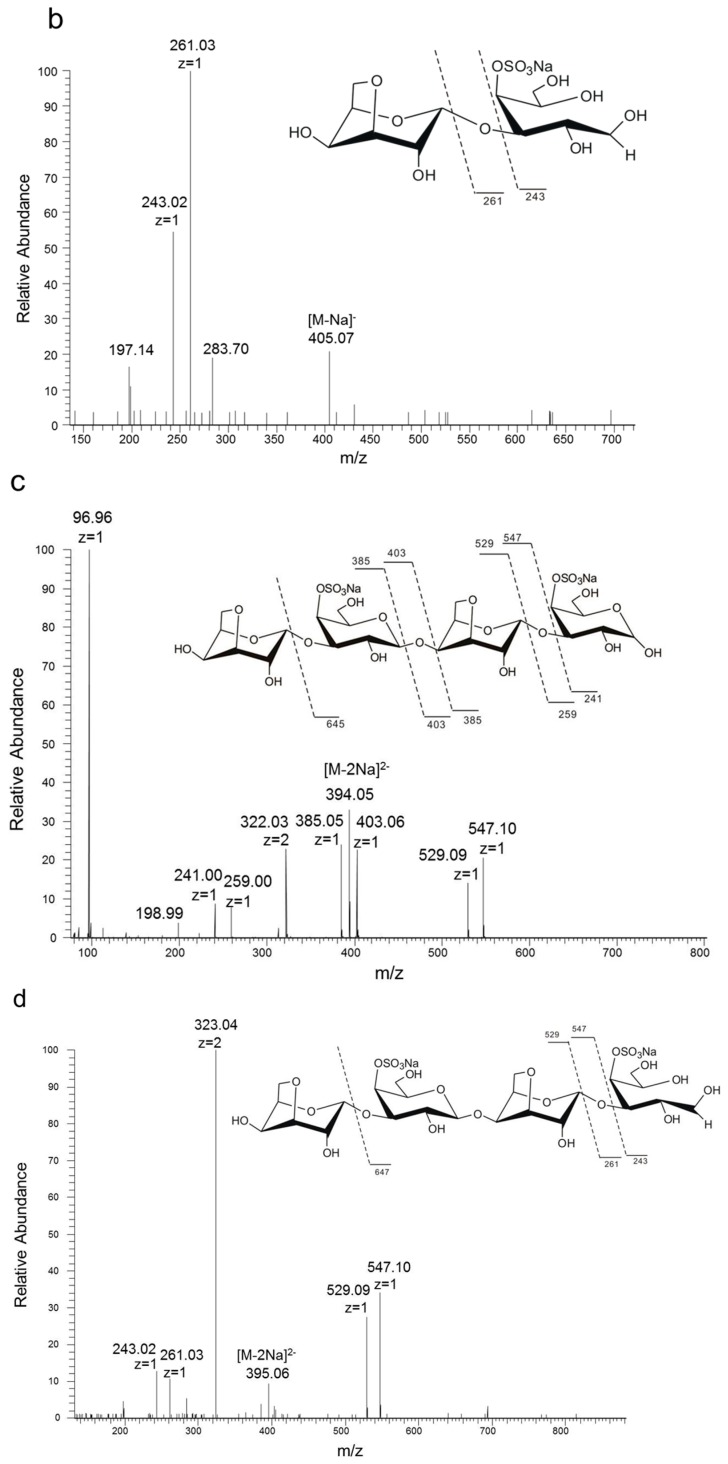
Electrospray ionization collision-induced-dissociation mass spectrometry (ESI-CID-MS^2^) spectrums of products of K-DP17 degraded by 38F6. (**a**) ESI-CID-MS^2^ spectrum of disaccharides generated from K-DP17 degradation by 38F6, (**b**) ESI-CID-MS^2^ spectrums of reduced disaccharides generated from K-DP17 degradation by 38F6, (**c**) ESI-CID-MS^2^ spectrums of tetrasaccharides generated from K-DP17 degradation by 38F6, (**d**) ESI-CID-MS^2^ spectrums of reduced tetrasaccharides generated from K-DP17 degradation by 38F6.

In order to analyze the enzyme hydrolyzing κ-carrageenan oligosaccharides in 38F6, the products generated by K-DP17 degradation after 144 h were separated using gel filtration and determined with electrospray ionization mass spectrometry (ESI-MS). The degradation products were identified mainly as 4-*O*-sulfate-d-galactose, κ-carratriose, κ-carrapentaose, κ-carraheptaose, and also a minor part was disaccharides and tetrasaccharides of κ-carrageenan (Table 1). The sequence of odd oligosaccharides can be easily verified based only on the results of ESI-MS for the different molecular weight of G4S and A (Table 1); however, for even oligosaccharides, there are two kinds of sequences, carraoligosaccharides (G4S-A-(G4S-A)_n_) and neocarraoligosaccharides (A-G4S-(A-G4S)_n_), and their molecular weights are the same. ESI-MS could not distinguish the difference between them according to mass spectrometry (MS); however, MS^2^ plus with oligosaccharide reduction could help to solve this problem. The *m*/*z* of ions generated from the reducing terminal fragments will increase after reduction, but there will be no change for fragments from non-reducing terminals [11]. Using neocarrabiose (A-G4S) as an example, *m*/*z* 259 (Y1) and 241 (Z1) (Figure 1a), which are from the reducing terminal, will become *m*/*z* 261 (Y1) and 243 (Z1) after reduction (Figure 1b); and based on the molecular weights, ions of *m*/*z* 259 and 241 should be from G4S, but not A, thus, G4S is at the reducing terminal. The electrospray ionization collision-induced-dissociation mass spectrometry (ESI-CID-MS^2^) spectrums of disaccharides (Figure 2a) and tetrasaccharides (Figure 2c) of κ-carrageenan from the degradation of K-DP17 showed that *m*/*z* 259 and 241 became *m*/*z* 261 and *m*/*z* 243 after reduction (Figure 2b,d). For tetrasaccharides, there is an additional ion, *m*/*z* 322 (double charged) (Figure 2c) became *m*/*z* 323 (double charged) (Figure 2d). All of the results indicated that the even-numbered oligosaccharides in the degradation production of K-DP17 are neocarrabiose (A-G4S) and neocarratetraose (A-G4S-A-G4S).

**Figure 3:**
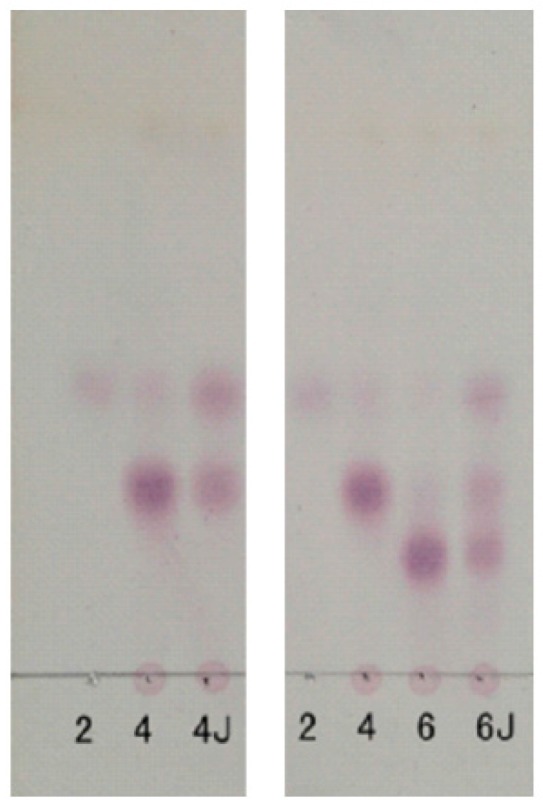
Thin-layer chromatography (TLC) analysis of degradation of neocarratetraose (NK-DP4) and neocarrahexaose (NK-DP6) by 38F6; 2: neocarrabiose (NK-DP2), 4: NK-DP4, 6: NK-DP6, 4J: NK-DP4 fermentation by 38F6, 6J: NK-DP6 fermentation by 38F6.

The degradation pattern of NK-DP4 and NK-DP6 also confirmed our inference. Thin-layer chromatography (TLC) results showed that the product of NK-DP4 is neocarrabiose (Figure 3), and of NK-DP6 are neocarrabiose and neocarratetraose (Figure 3), which further demonstrate that the enzyme secreted by 38F6 is β-carrageenase, specifically cleaving β-1,4-glycoside (Figure 6a).

### 2.2. Chemical Structures of the Intermediates Produced by the Degradation of AP by B. uniformis L8

**Figure 4:**
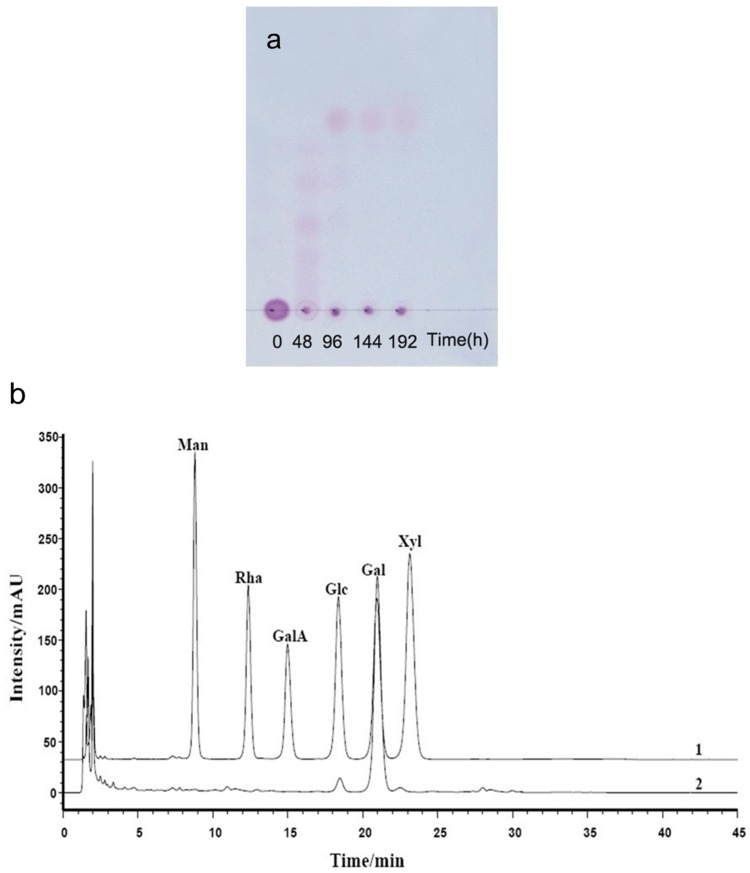
Analysis of the products of agarose (AP) degraded by *B. uniformis* L8 (**a**) TLC analysis of degradation of AP by *B. uniformis* L8 at 0, 48, 96, 144 and 192 h; (**b**) HPLC chromatography of final products of AP degraded by *B. uniformis* L8 at 96 h; 1. monosaccharide standard (Man: mannose, Rha: rhamnose, GalA: galacturonic acid, Glc: glucose, Gal: Galactose, Xyl: xylose); 2. final products of AP degraded by *B. uniformis* L8 at 96 h.

**Figure 5:**
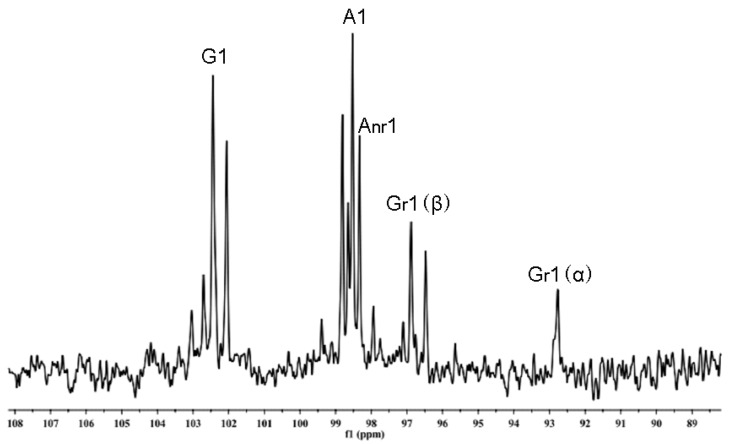
NMR ^13^C spectrum of the oligosaccharides generated from agarose (AP) degradation by *B. uniformis* L8 at 48 h.

TLC patterns showed that the intermediates produced from the degradation of AP by *B. uniformis* L8 contained a range of fragments with different molecular weights after 48 h of incubation (Figure 4a). However, the product generated after 96 h of incubation consisted of only a single spot (Figure 4a), which was identified as d-galactose by HPLC, using the 1-phenyl-3-methyl-5-pyrazolone (PMP)-derivatization method (Figure 4b). The chemical structures of the intermediates at 48 h were determined using ESI-MS analyses and NMR. The ESI-MS results showed that the composition of the intermediates from AP degradation by *B. uniformis* L8, after 48 h, mainly included disaccharides, tetrasaccharides, hexasaccharides, and octasaccharides of agarose (Table 2). Agarose is made up of 3, 6-anhydro-l-galactose (A) and d-galactose (G) units, alternately linked by α-(1, 3) and β-(1, 4) glycosidic bonds. Like κ-carrageenan oligosaccharides, there are still two different sequences for the oligosaccharides of agarose: Agaroligosaccharides (G-A-(G-A)_n_) and neoagaroligosaccharides (A-G-(A-G)_n_); ESI-MS could not tell the difference between these two sequences, thus, part of the degradation products at 48 h was taken for NMR analysis to determine the sequence. Compared to the standard spectrums of agaroligosaccharides and neoagaroligosaccharides, the signals, 92.8 ppm, 96.8 ppm, and 98.3 ppm, were, respectively, ascribed to the G_r_1(α), G_r_1(β), and A_nr_1 residues, which were exactly in accord with the typical anomeric carbon signals of neoagaroligosaccharides [12] (Figure 5 and Table 3).

**Figure 6:**
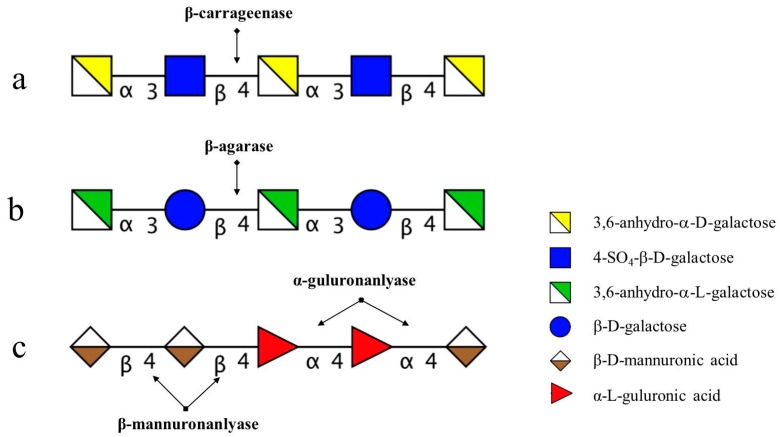
Profile of degradation position and mode of action of enzymes on marine carbohydrates. (**a**) degradation position of carrageenan oligosaccharides by enzymes secreted by 38F6 (*B. xylanisolvens* and *E. coli*); (**b**) degradation position of agarose (AP) by enzymes from *B. uniformis* L8; (**c**) degradation position of alginate by enzymes from *B. ovatus* G19.

There are two types of agarase: α-agarase and β-agarase. α-agarase cleaved α-1,3-glycosidic bonds, releasing agaroligosaccharides (G-A-(G-A)_n_), on the other hand, β-agarase cleaved β-1,4-glycosidic bonds and the degradation products are neoagaroligosaccharides (A-G-(A-G)_n_). Considering the results of ESI-MS and NMR, the major secreted enzyme of *B. uniforms* L8 should be β-agarase, which specifically cleaves the β-1,4-glycosidic bonds between G and A (Figure 6b). However, the end product of AP degradation by *B. uniforms* L8 is d-galactose, so it is possible that during the later period of hydrolysis, *B. uniforms* L8 secreted another glycoside hydrolase, which could help to hydrolyze neoagarobiose to D and A. The reason that 3,6-anhydro-galactose was not detected during the PMP-derivatization method is that it was further degraded to 5-hydroxymethyl-furfural due to its instability [13].

### 2.3. Chemical Structures of the Intermediates Produced by the Degradation of Alg, MO and GO by B. ovatus G19 

Alginate consists of hexuronic acid residues, β-d-mannuronic acid (M), and α-l-guluronic acid (G) with only 1→4 glycosidic linkages. MO and GO used for digestion were obtained from acid hydrolysis; controlled acid hydrolysis results in random cleavage along the polysaccharide chains and produces oligosaccharide fragments with unmodified hexuronic acid residues at both termini, and there are no unsaturated hexuronic acid residues in the products from acid hydrolysis. Alginate lyase are classified as α-1,4-guluronanlyase and β-1,4-mannuronanlyase; some bacteria can only secrete one kind of alginate lyase [14,15], and there are also bacteria that can secrete both [16]. All of the oligosaccharides generated from the digested enzyme had the 4,5-unsaturated hexuronic acid residue (∆) at the non-reducing terminus. 

The degradation productions of Alg, MO, and GO were prepared following the above-mentioned method. Fractions of C1, C2, C3, and C4 were obtained from degradation products of Alg and the procedure was performed on a LTQ Orbitrap XL instrument in order to determine molecular weight. Based on the molecular weight from the ESI-MS results (Table 4), C1 to C4 were assigned as unsaturated alginate disaccharides, trisaccharides, tetrasccharide, and pentasaccharides. MO and GO could also be digested by *B. ovatus* G19; the products contained saturated and unsaturated disaccharides, trisaccharides, and tetrasccharide (data not shown). According to *B. ovatus* G19’s degrading ability of Alg, MO, and GO, the enzymes from *B. ovatus* G19 contain both α-1,4-guluronanlyase and β-1,4-mannuronanlyase, resulting in unsaturated alginate oligosaccharides (Figure 6c). For MO and GO, because the original substrate was prepared using acid hydrolysis, the saturated oligosaccharides are from the hexuronic acid residues at the non-reducing end.

## 3. Experimental Section 

### 3.1. Polysaccharide and Oligosaccharide Materials

AP was obtained from Qingdao Judayang Seaweed Co. Ltd., Qingdao, China. Other marine oligosaccharides used in the current study, including several kinds of κ-carrageenan oligosaccharides: NK-DP4, NK-DP6, and K-DP17, were kindly provided by Glycoscience and Glycoengineering Laboratory, Ocean University of China. GO and MO were obtained from Qingdao Haida Science and Technology Pharmaceutical Company (Qingdao, China). The purity of MO (with a molecular weight of 2.5 kD) and GO (with a molecular weight of 4.0 kD) were at least 90%, based on monosaccharide analyses using pre-column derivation with PMP by HPLC. 

### 3.2. Bacteroidetes Material

Bacteroidetes responsible for degrading these marine carbohydrates were all isolated from human fecal samples and identified by sequencing their 16S rRNA gene. *Bacteroides uniforms* L8 could degrade agaro-oligosaccharides [8]; *Bacteroides xylanisolvens* and *E. coli* (38F6) were identified for degrading κ-carrageenan oligosaccharides mixture [10]; and *Bacteroides ovatus* G19 was reported to degrade alginate and alginate oligosaccharides [9]. 

### 3.3. Degradation of Marine Carbohydrates by Human Gut Bacteria Isolates

Batch culture fermentations were conducted using the procedure described by Lei et al. [17]. Briefly, the basic growth medium VI contained the following (g/L): Yeast extract, 4.5; tryptone, 3.0; peptone, 3.0; bile salts No. 3, 0.4; l-cysteine hydrochloride, 0.8; NaCl, 4.5; KCl, 2.5; MgCl_2_·6H_2_O, 0.45; CaCl_2_·6H_2_O, 0.2; KH_2_PO_4_, 0.4; Tween 80, 1 mL; and 2 mL of a solution of trace elements. Different marine carbohydrates were added to VI separately at different concentrations for their own properties: NK-DP4, NK-DP6, K-DP17, MO, GO, 8 g/L; AP, 1 g/L; Alg, 5 g/L. Medium with κ-carrageenan oligosaccharides (NK-DP4, NK-DP6 and K-DP17), AP, and alginate type (Alg, GO, MO) were autoclaved and inoculated with 38F6, *B. uniforms* L8, and *Bacteroides ovatus* G19 separately, then incubated at 37 °C in an anaerobic chamber. Samples were removed at different times for analyses of degradation.

### 3.4. General Experimental Procedures

The derivatives generated from NK-DP4, NK-DP6, and AP were confirmed using TLC analysis. Samples (0.2 μL) were loaded on a pre-coated silica gel-60 TLC aluminum plates (Merck, Darmstadt, Germany). After development with a solvent system consisting of formic acid/n-butanol/water (6:4:1, *v*/*v*/*v*), the plate was soaked in orcinol reagent and visualized by heating at 120 °C for 3 min.

The derivatives generated from K-DP17, AP, Alg, MO, and GO with Bacteroidetes were determined by gel filtration chromatography and analyzed using negative-ion ESI-MS for K-DP17, Alg, MO and GO; positive-ion ESI-MS for AP. In brief, after removing the bacteria by centrifugation, the supernatant was separated on a Superdex Peptide 10/300 column [18] and the sequence of each fraction was determined on a Thermo LTQ Orbitrap XL instrument (Thermo Fisher Scientific, Waltham, MA, USA) [13,19,20]. Samples were dissolved in CH_3_CN/H_2_O (1:1, *v*/*v*) at a concentration of 10 pmol/μL and 5 μL was injected. Solvent volatilization temperature and capillary temperatures were 275 °C and the sheath flow gas flow rate was 8 arb. The flow rate was 8 μL/min in the ESI-MS analysis and 3–5 μL/min in the ESI-CID-MS^2^ analysis. Helium was used as the collision gas with a collision energy of 20–25 eV.

Because TLC analyses showed only one spot of AP after 96 h of incubation with *B. uniforms* L8, monosaccharide analysis was performed using pre-column derivation with PMP [21]. Briefly, the end products of AP were derivatized with PMP and then analyzed using HPLC system (Agilent 1260, Santa Clara, CA, USA) on a XDB-C18 column with acetonitrile/phosphate buffer solution (18:82, pH 6.7) at a flow rate of 1.0 mL/min at 30 °C; the detection wavelength was set to 254 nm. The composition of the end products was determined by retention time, in comparison with monosaccharide standards (mannose, rhamnose, xylose, galactose, glucose, and glucuronic acid; Sigma-Aldrich Company, Shanghai, China).

Part of the AP degradation solution after centrifugation was precipitated with 2 volumes of ethanol, twice, and then dried. Thirty milligrams of dried sample were co-evaporated by lyophilization, twice, with 1 mL of D_2_O (99.96%), to remove exchangeable protons before a final dissolution in 0.5 mL of D_2_O for NMR analysis. ^13^C-NMR spectra were acquired at 25 °C with a JEOL ECP 600 MHz spectrometer (JEOL, Tokyo, Japan). Chemical shift values were calibrated using acetone-d66 as an internal standard.

### 3.5. Oligosaccharide Reduction

In order to analyze the sequence of products generated from K-DP17 degradation, disaccharides and tetrasaccharides of carrageenan (20–50 μg) were added 20 μL of NaBD_4_ reagent (0.05 M NaBD_4_ in 0.01 M NaOH) and the reduction was carried out at 4 °C overnight, as previously described [22]. The reaction solution was then neutralized to pH 7 with a solution of AcOH/H_2_O (1:1) to damage borodeuterides before passing through a mini-column of cation exchange (AG50W-X8, Bio-Rad, Hercules, CA, USA). Boric acid was removed by repeated co-evaporation with MeOH.

## 4. Conclusions

Our study showed that marine carbohydrates (carrageenan, agarose, alginate, and their oligosaccharide derivatives), which could not be digested by humans, can be degraded by specific Bacteroidetes isolated from human gut microbiota; we also analyzed the enzymes responsible for hydrolysis, secreted by these Bacteroidetes. It was reported that all of the oligosaccharides of carrageenan, agarose, and alginate showed many potential activities with respect to antiviral [23], anticancer [24], and hypolipidemic effects [25]. Thus, the oligosaccharides generated from the degradation have the potential to affect the structure of human gut microbiota, and also gut health. Further animal studies are required to evaluate the effects of these marine carbohydrates, combined with degrading Bacteroidetes in organisms.

## Figures and Tables

**Table 1:** Negative-ion electrospray ionization mass spectrometry (ESI-MS) analysis of the fragments generated from K-DP17 degradation by 38F6 at 144 h.

Fractions	Found Ions (Charge)	Calculated Mol Mass (Na Form)	Assignment	
			DP	Sequences
A 1	403.06 (−1)	426.05	2	A-G4S
A 2	322.03 (−2)	690.05	3	G4S-A-G4S
A 3	394.05 (−2)	834.10	4	A-G4S-A-G4S
A 4	343.03 (−3)	1098.09	5	G4S-A-G4S-A-G4S
A 5	391.06 (−3)	1245.18	6	A-G4S-A-G4S-A-G4S

G4S: (1→3)-4-SO_4_-β-d-galactose; A: (1→4)-3,6-anhydro-α-d-galactose.

**Table 2:** Positive-ion electrospray ionization mass spectrometry (ESI-MS) analysis of the fragments generated from agarose (AP) degradation by *B. uniformis* L8 at 48 h.

Fraction	Found Ions (Charge)	Calculated Mol Mass (H Form)	Assignment	
			DP	Sequences
B1	325.11 * (+1)	324.11	2	A-G
B2	653.19 ** (+1)	630.20	4	A-G-A-G
B3	959.29 ** (+1)	936.30	6	A-G-A-G-A-G
B4	1265.38 ** (+1)	1242.39	8	A-G-A-G-A-G- A-G

G: (1→3)-β-d-galactose; A: (1→4)-3,6-anhydro-α-l-galactose; * the found ion is H form; ** the found ion is Na form.

**Table 3:** NMR ^13^C spectrum ascription of standard agarose oligosaccharides and products generated from agarose (AP) degraded by *B. uniformis* L8 at 48 h.

Compound	G1	G_r_1	G_nr_1	A1	A_r_1	A_nr_1
neoagaroligosaccharides ^a^	102.4	92.8(α) 96.8(β)	-	98.5	-	98.3
neoagarotetraose ^a^ A_nr_-G_nr_-A_r_-G_r_	102.4	92.8(α) 96.8(β)	-	98.5	-	98.3
agaroligosaccharides ^b^	102.6	-	102.6	98.4	90.4	
agarotetraose ^b^ G_nr_-A_nr_-G_r_-A_r_	102.6	-	102.5	98.5	63.3	
AP-L8	102.44	92.77(α) 96.88(β)		98.53	-	98.33

G: (1→3)-β-d-galactose; A: (1→4)-3,6-anhydro-α-l-galactose; r: reducing end; nr: non-reducing end; a: agarose oligosaccharides generated from agarose hydrolyzed by β-agarase; b: agarose oligosaccharides generated from agarose hydrolyzed by α-agarase.

**Table 4:** Negative-ion electrospray ionization mass spectrometry (ESI-MS) analysis of the fragments generated from alginate (Alg) degradation by *B. ovatus* G19 at 144 h.

Fraction	Found Ions (Charge)	Calculated Mol Mass (H Form)	Assignment	
			DP	Sequences
C1	351.05 (−1)	351.06	2	∆NN
C2	527.08 (−1)	527.09	3	∆NNN
C3	351.05 (−2), 703.11 (−1)	704.13	4	∆NNNN
C4	439.06 (−2), 879.13 (−1)	880.16	5	∆NNNNN

N = β-d-mannuronic acid (M) or α-l-guluronic acid (G).
